# Stress and stability of plate-screw fixation and screw fixation in the treatment of Schatzker type IV medial tibial plateau fracture: a comparative finite element study

**DOI:** 10.1186/s13018-015-0325-2

**Published:** 2015-11-25

**Authors:** Xiaowei Huang, Zhongzheng Zhi, Baoqing Yu, Fancheng Chen

**Affiliations:** Shanghai Medical College, Fudan University, Shanghai, China; Department of Orthopedics, Shanghai Pudong Hospital, Fudan University Pudong Medical Center, No. 2800 Gongwei Road, Huinan Town, Pudong New Area, Shanghai City, China

**Keywords:** Finite element analysis, Medial tibial plateau fracture, Schatzker IV, Comparison

## Abstract

**Purpose:**

The purpose of this study is to compare the stress and stability of plate-screw fixation and screw fixation in the treatment of Schatzker type IV medial tibial plateau fracture.

**Methods:**

A three-dimensional (3D) finite element model of the medial tibial plateau fracture (Schatzker type IV fracture) was created. An axial force of 2500 N with a distribution of 60 % to the medial compartment was applied to simulate the axial compressive load on an adult knee during single-limb stance. The equivalent von Mises stress, displacement of the model relative to the distal tibia, and displacement of the implants were used as the output measures.

**Results:**

The mean stress value of the plate-screw fixation system was 18.78 MPa, which was significantly (*P* < 0.001) smaller than that of the screw fixation system. The maximal value of displacement (sum) in the plate-screw fixation system was 2.46 mm, which was lower than that in the screw fixation system (3.91 mm). The peak stress value of the triangular fragment in the plate-screw fixation system model was 42.04 MPa, which was higher than that in the screw fixation model (24.18 MPa). But the mean stress of the triangular fractured fragment in the screw fixation model was significantly higher in terms of equivalent von Mises stress (EVMS), *x*-axis, and *z*-axis (*P* < 0.001).

**Conclusions:**

This study demonstrated that the load transmission mechanism between plate-screw fixation system and screw fixation system was different and the stability provided by the plate-screw fixation system was superior to the screw fixation system.

## Introduction

Tibial plateau fractures account for approximately 9.2 % of all tibia fractures [1, 2], which involve the articular surface of the proximal tibia that supports the opposing femoral condyle. As tibial plateau fractures are often caused by high-energy trauma with displaced fractured fragments, surgical approaches are often needed to maintain anatomical reduction and to prevent the development of devastating complications [3].

To maintain the stability of the fractured tibial plateau, various approaches have been developed. Locked plates with open reduction and internal fixation (ORIF) techniques and percutaneous lag screws with arthroscopy-assisted reduction and fixation techniques are the two most frequently used surgical approaches. The former provides stability to the fractured tibial plateau by locking head screws and the contour of the plate itself, and the lag screws maintain their fixation through compression and buttressing effect with lesser soft-tissue dissection [4]. However, the comparative biomechanics between these two different implants has not been demonstrated and analyzed computationally.

Finite element analysis is one of the computational methods that have received wide acceptance in the field of orthopedic research, where three-dimensional models of bone-implant construct are converted into finite elements with the application of simulated physiological loads to analyze and predict the outcome of surgery [5]. Biomechanical studies via computational simulation can provide deeper insight into the stability and functionality of bone constructs [6]. Therefore, the present study was conducted to determine the stability of two surgical approaches aforementioned to treat one type of tibial plateau fracture (Schatzker type IV) through the use of finite element analysis.

## Materials and methods

This study was approved by the Institutional Review Board of the Shanghai Pudong hospital. Written informed consent from the subject was obtained prior to his taking part in the study. After excluding comorbidities such as osteoarthritis, pelvic, and lower-extremity fractures, a 35-year-old healthy male with a weight of 75 kg and a height of 170 cm underwent a CT scan of his left lower limb.

### Experimental model

**Fig. 1:**
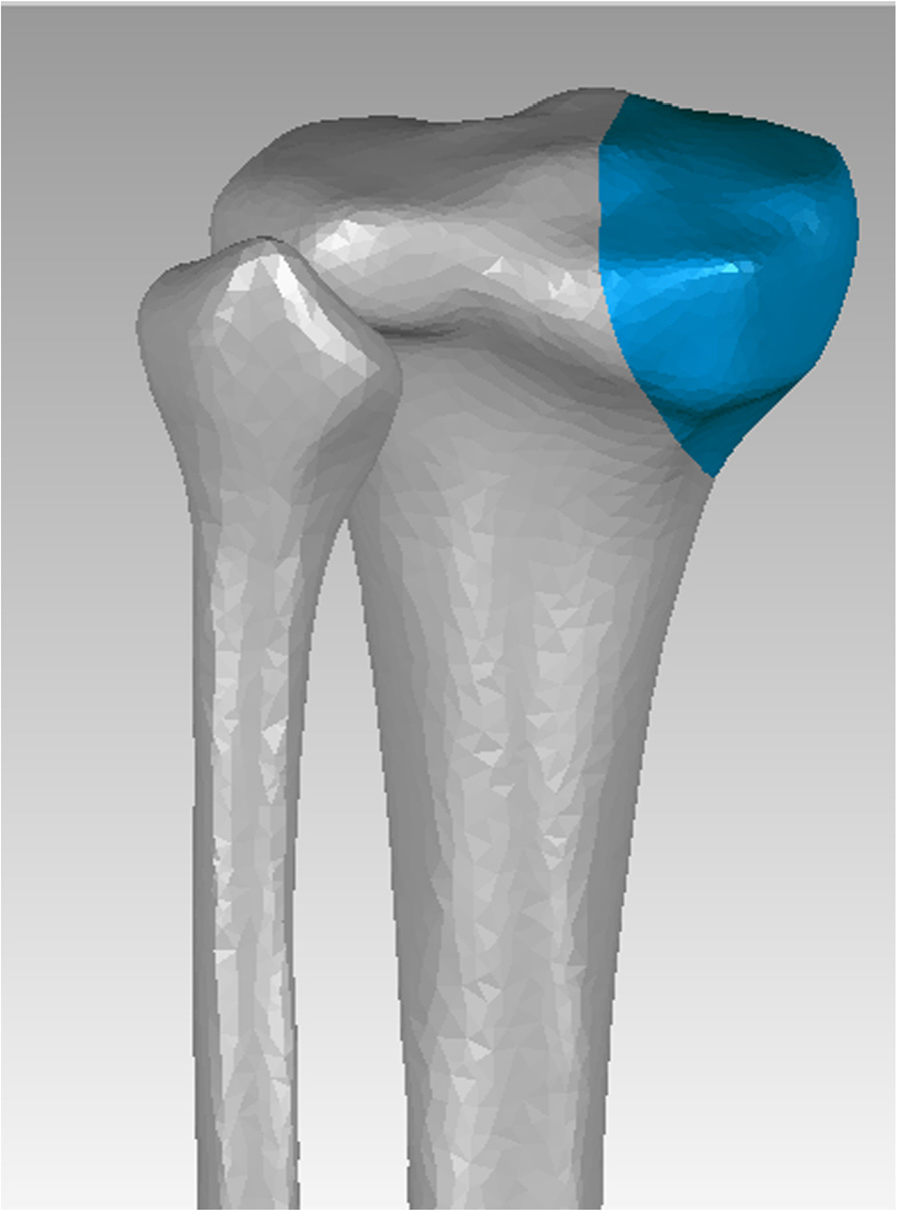
The creation of fracture line

Initial CT data of the tibia and fibula were obtained with 1-mm cuts from the selected male subject. Three-dimensional (3D) models of the human tibia and fibula were reconstructed from the CT data in the Digital Imaging and Communications in Medicine (DICOM) format using Mimics software (Materialize Company, Leuven, Belgium). Then, the previously built model was imported to Geomagic Studio Software (3D system Inc., Rock Hill, SC, USA) for further polishing and to create the fracture line. The medial plateau fracture (Schatzker type IV fracture) was simulated in the model with an oblique cut starting from 5 cm distal to the joint line of the medial tibial plateau and extending to the medial border of the tibial tubercle [7], as shown in Fig. 1. No displacement was created between the two fractured segments. The 3D model of the screws and plate was created according to the specifications of the manufacturer using computer-aided design software. The plate-screw system was comprised of seven screws, each with a diameter of 3.5 mm (four cancellous screws placed proximally and three cortical screws placed distally), and one T-shaped plate with a thickness of 4 mm. The most proximal three cancellous screws and the most distal two cortical screws were parallel to the joint surface, while the other two screws were at 20° and 30° angles to the joint surface. Three cancellous screws with a 16-mm partial thread and a diameter of 6.5 mm made up the screw fixation system (two proximal screws placed parallel to the joint surface and one proximal screw placed at a 20° angle to the joint surface). The distance between the proximal screws was 3.5 cm, and the screws were inserted 1 cm distal to the joint. The third screw was inserted into the apex of the triangular fragment. The 3D model of the T-shaped plate was created on the basis of a T-shaped surface cut from the tibia model to make the plate perfectly fit to the tibia with the utilization of reverse engineering techniques. Then, the model was imported to ANSYS software for re-meshing and subsequent establishment of the finite element model. In this study, an eight-node hexahedron three-dimensional element was utilized in the selection of the unit type because it is more suitable for geometric non-linear analysis than second-order elements. The number of elements and nodes in each component are listed in Table 1.

**Table 1:** The comparison of stress and displacement between two models and the number of nodes and elements in each model (The model fixed with T-shaped plate was designated as ‘P’ and the model fixed with three percutaneous screws was designated as ‘S’. The data was listed in the form of ‘mean ± SD; number of nodes’.)

Variable	Model P	Model S	*P* value
Stress on implants (MPa)	18.78 ± 22.29; 5714	40.79 ± 48.59; 2820	<0.001
Stress on tibia and fibula (MPa)	3.79 ± 5.59; 35,144	5.82 ± 7.68; 32,653	<0.001
Displacement of models (*x*-axis) (mm)	0.49 ± 0.31; 38,511	1.09 ± 0.66; 26,994	<0.001
Displacement of models (*y*-axis) (mm)	1.97 ± 0.82; 38,511	2.10 ± 1.12; 26,994	<0.001
Displacement of models (*z*-axis) (mm)	0.25 ± 0.17; 38,511	0.32 ± 0.38; 26,994	<0.001
Displacement of models (sum) (mm)	1.77 ± 0.80; 38,511	2.40 ± 1.31; 26,994	<0.001
Stress on the triangular fractured fragment (EVMS) (MPa)	2.54 ± 4.28; 3473	3.79 ± 3.57; 3071	<0.001
Stress on the triangular fractured fragment (*x*-axis) (MPa)	2.16 ± 0.01; 3473	2.72 ± 3.55; 3071	<0.001
Stress on the triangular fractured fragment (*y*-axis) (MPa)	0.63 ± 1.10; 3473	0.34 ± 1.05; 3071	<0.001
Stress on the triangular fractured fragment (*z*-axis) (MPa)	−0.82 ± 2.03; 3473	−1.67 ± 2.22; 3071	<0.001
Model	Number of nodes	Number of elements	N/A
Model P	38,511	206,342	N/A
Model S	26,994	143,455	N/A

N/A means not applicable in Table 1

### Material properties

In this study, the properties of titanium alloy were assigned to the simulated implant models, with an elastic modulus of 110 GPa and a Poisson ratio of 0.3 [8, 9]. It was presumed that both implants and bones were isotropic, linear, and elastic materials. An elastic modulus of 17 GPa with a Poisson’s ratio of 0.33 and an elastic modulus of 5 GPa with a Poisson’s ratio of 0.33 were chosen for the cortical bone and the trabecular bone, respectively [10].

### Loading and boundary conditions

An axial force of 2500 N with a distribution of 60 % to the medial compartment was applied to simulate the axial compressive load on an adult knee during single-limb stance [8, 11]. For both the plate-screw and screw fixation systems, it was assumed that the implants and bones were in direct contact with a frictional coefficient of 0.3 [12]. The interaction between the plate and screw was simulated to imitate strong contact attachment by sharing the common nodes of elements to mimic the locking screw mechanism. The effect of load sharing between the tibia and fibula was simulated by the bonding effect.

### Analysis

The analysis was performed using ANSYS software. In this study, the equivalent von Mises Stress (EVMS), displacement of the model relative to the distal tibia, and displacement of the implants were used as the output measures. For statistical analysis, Student’s *t* test was used to compare the mean values of stress and displacement between the two models [8]. A *P* value less than 0.05 was regarded as statistically significant.

## Results

### Stress on two implants

**Fig. 2:**
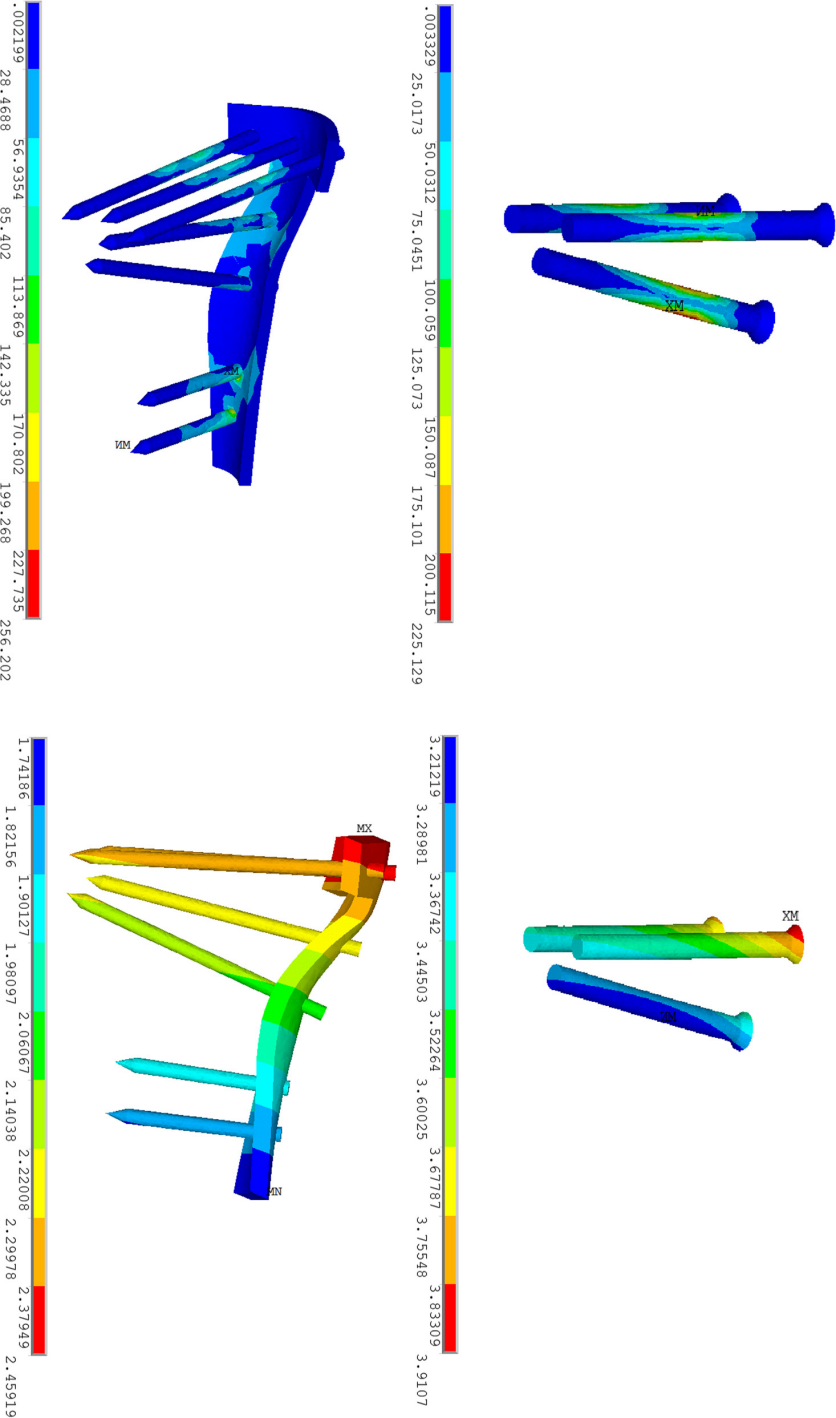
Stress distribution (*upper*) and displacement pattern (*lower*) on implants

The stress distribution in the plate-screw system was different from that in the screw fixation system. The maximal stress value in the plate-screw system was 256.20 MPa, while that of the screw fixation system was 225.12 MPa, as shown in Fig. 2. In the plate-screw system, maximal stress concentrated on the intersection area between the second distal screw and the plate, as shown in Fig. 2. In the screw fixation system, however, the maximal stress was found in the middle section of the most distal screw. Similarly, stress concentration was observed on the middle section of the four proximal screws in the plate-screw system as well. Stress concentration was also obvious in the proximal part of the T-shaped plate that was in direct contact with the proximal triangular fragment and the fracture line. At the distal end of the plate, stress also concentrated on the distal plate and in the intersection area between the plate and the screws. The mean stress value, which was calculated from the stress of all of the nodes within the plate, was also compared between the two fixation systems, as listed in Table 1. The mean stress value of the plate-screw system was 18.78 MPa, which was significantly (*P* < 0.001) smaller than that of the screw fixation system which had a value of 40.79 MPa.

### Displacement of two models

**Fig. 3:**
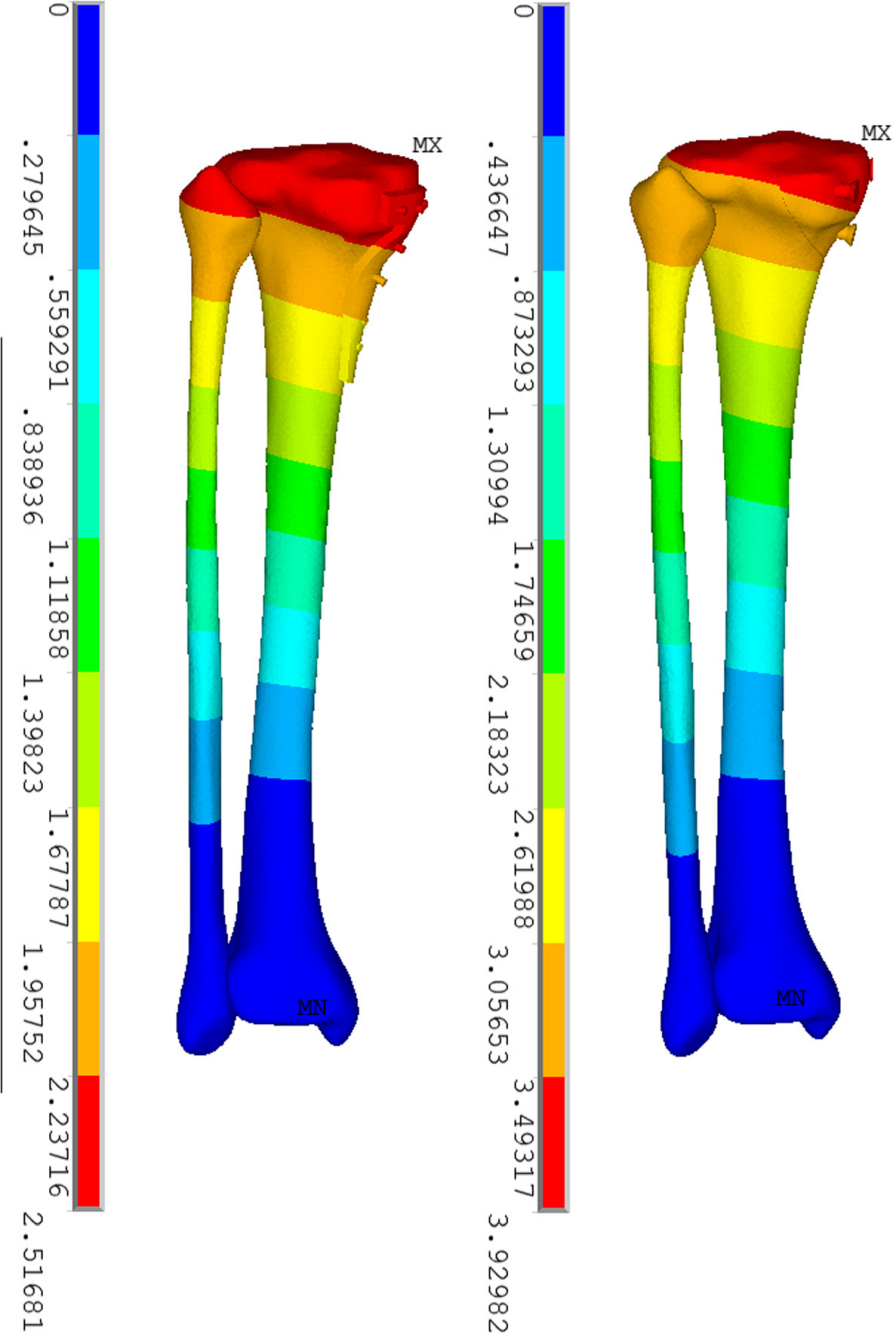
Displacement pattern on the two models fixed with plate-screw system (*left*) and screw fixation system (*right*)

It appeared that higher amounts of displacement were observed in the model with the screw fixation system, as illustrated in Fig. 3. The maximal displacement (sum) in the plate-screw fixation model was 1.77 mm, which was smaller than that in the plate-screw model, which was 2.40 mm. Additionally, there were statistically significant differences in the mean displacement between the two models in the three different axes, respectively, (*P* < 0.001), as shown in Table 1. In addition, the maximal value of displacement (sum) in the plate-screw system was 2.46 mm, which was lower than that in the screw fixation system (3.91 mm). Both of the two models shared a similar trend of the displacement decreasing as it approaches distally.

### Stress on the fractured surface and the triangular fragment

**Fig. 4:**
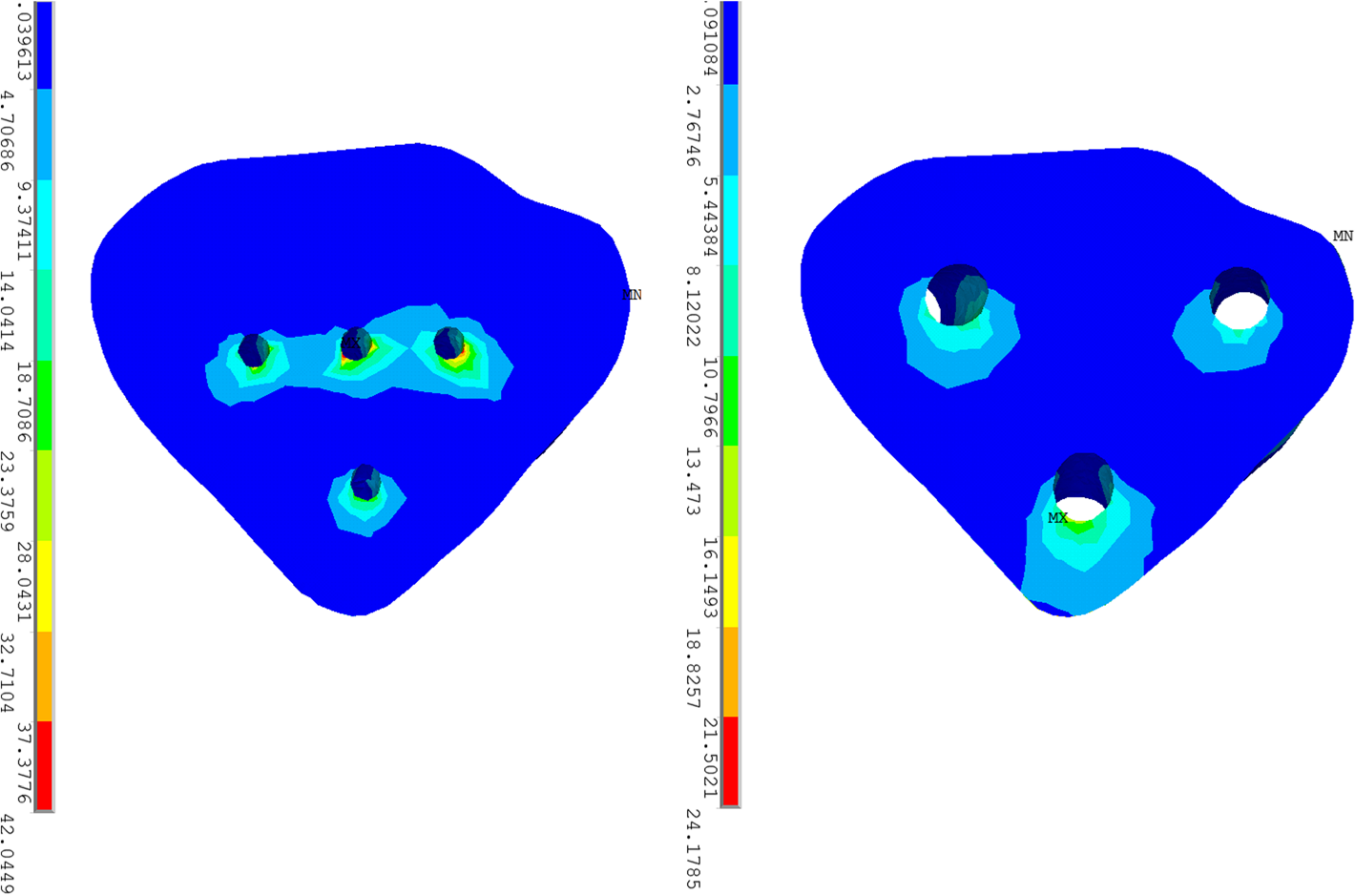
Stress distribution on the small triangular fragment in plate-screw model (*left*) and screw fixation model (*right*)

The distribution of stress on the fractured surface and the small triangular fragment was compared and is illustrated in Fig. 4. The pattern of stress concentration on the fractured surface, which was located surrounding the screw holes and on the distal end of the fractured surface, was similar for the two models. The peak stress value of the triangular fragment in the plate-screw system model was 42.04 MPa, which was higher than that in the screw fixation model (24.18 MPa). However, when taking all the nodes of the triangular fragment into consideration, the mean stress of the triangular fractured fragment in the screw fixation model was significantly higher in terms of EVMS, *x*-axis, and *z*-axis, as listed in Table 1. The maximal stress was mainly located near the distal hole on the screw fixation model, while the proximal screw holes experienced the maximal stress on the plate-screw fixation model.

## Discussion

Medial tibial plateau fractures are often caused by high-energy trauma and are relatively uncommon compared with lateral tibia plateau fractures [13]. They are usually more unstable because the medial tibial plateau is subjected to higher loads [14] and lacks buttress structures such as the fibula head to support the lateral tibial plateau [7]. Though favorable outcomes have been reported concerning the utilization of percutaneous cancellous screws with arthroscopically assisted techniques in the treatment of lateral tibia plateau fractures [4], the feasibility of this method to treat medial tibial plateau fractures still needs to be verified. In the paper, a study of comparison between the plate-screw fixation system which utilized the locking compression plate design and the screw fixation system which included three cancellous lag screws for the treatment of medial tibial plateau fracture was performed.

The characteristics of the stress distribution on the plate-screw and screw fixation models were analyzed through EVMS. It was observed that the stress concentration on the plate-screw system was located on the proximal part of the T-shaped plate that was in direct contact with the proximal triangular fragment and the fracture line, indicating the anti-sliding/buttress effect provided by the plate. Additionally, the mean stress on the bones (both the tibia and the fibula) was significantly smaller than that on the bones fixed with screw fixation system, as shown in Table 1. This can be explained by the fact that the T-shaped plate with perfect contouring provides better anchorage and load sharing. As the plate-screw system replaced the medial cortex as the main path of loading transmission, the mean stress on the triangular fractured fragment was smaller than that in the screw fixation system. In addition, the stress concentration was found on the distal part of the plate particularly on the regions surrounding the screw holes. A similar concentration of stress was also observed on the Tomofix plate system used to maintain the medial open wedge [8]. Although loading transmission through the plate-screw system may provide better stability, the accompanying stress shielding effect can cause severe osteoporosis and can increase the risk of re-fracture [15, 16]. Additionally, comparatively higher maximal stress in the plate-screw system may increase the risk of implant failure. However, without the normal data that determines the threshold to cause implant failure or for re-fracture to occur, these concerns may be unwarranted.

**Fig. 5:**
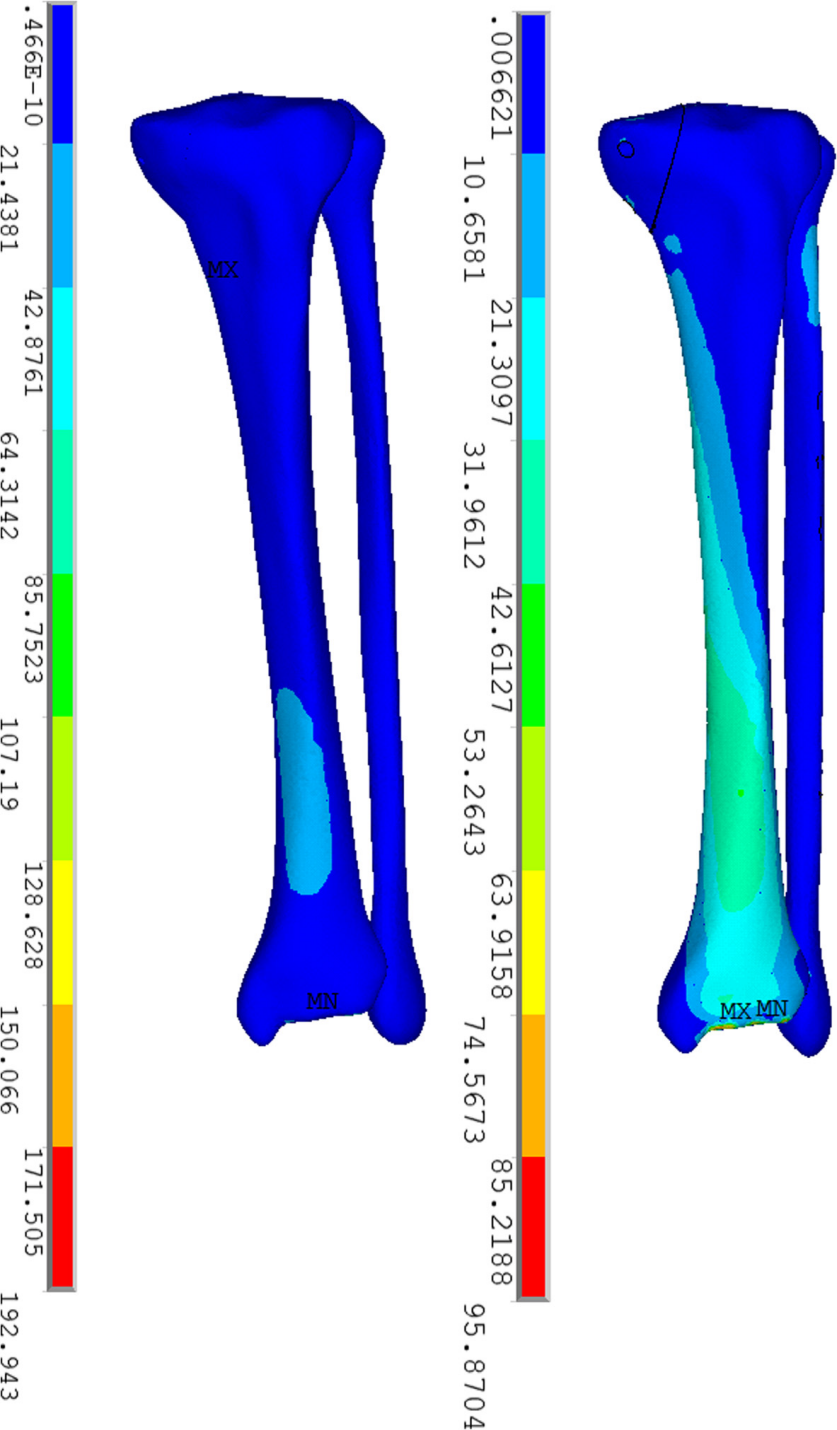
Stress distribution on the plate-screw model (*left*) and screw fixation model (*right*) with the exclusion of implants

It is interesting to note that the stress distribution on the screws of the two models shared a similar tendency. The stress concentrated on the middle section of the four proximal screws in the plate-screw system and on three screws in the screw fixation system, which just went across the fracture line and were the main part that provided the buttress and anti-sliding function. In both models, stress concentrated on the anterior distal part of the tibia (Fig. 5). This finding has been reported in previous studies and was attributed to the slenderness of the tibia itself [17–19].

Excessive displacement of implants may result in loosening and compromise the stability of fixation, as reported previously by several studies [19]. Excessive displacement of the whole model relative to the distal end may also increase the risk of implant failure [20]. In the present study, the mean displacement of the screw fixation model was relatively higher than that of the plate-screw fixation model. The maximal displacement of the screws was also comparatively higher than that in the plate-screw system. These findings indicated that the plate-screw fixation system provided better stability.

However, the screw fixation system also had its merits. The stress distributed more evenly on both implants and bones. The maximal stress on the implant and the triangular fragment was smaller than in the screw fixation system, which was attributed to the larger loading area on the percutaneous screws. In general, the screw fixation system distributed the stress more evenly and did not interrupt the loading transmission via the medial tibial plateau, which may be preferred for the fixation in young patients whose bones are biomechanically sound and do not have osteoporosis.

Only a few biomechanical studies focusing on the comparison of screw-plate versus screw fixation in the treatment of tibia plateau fractures have been released. Cift et al. [7] compared the efficacy of plate-screw fixation and screw fixation with an artificial bone model to imitate a Schatzker type IV fracture. After axial compression loading was applied, it was found that the load-bearing capacity of the model fixed by plate-screw fixation (2153.2 ± 204.4 N) was significantly higher than that of the model fixed with three cancellous screws (1397.6 ± 194.4 N), which are in agreement with the results of our present study. Boisrenoult et al. [21] compared the capability of screw-plate fixation with double-screw fixation on a non-embalmed cadaver model with a Schatzker type II fracture and found no significant difference in the biomechanical stability. The inconsistence of results in the two studies may be explained by the fact that the lateral buttress structures, such as the fibular head, can strengthen the stability of lateral tibial plateau [7]. Unlike the two biomechanical studies aforementioned where the bone-implant construct was taken as a whole, the present study via finite element analysis can demonstrate the stress and displacement pattern of implants and bones separately, which makes the analysis of each component possible.

In this study, with the intention to imitate the anatomical reduction of the medial tibial plateau fracture, no displacement between the fractured segments was created, which made the model the same as a simple non-displaced fracture. However, it should be noted that multiple pinning under the guidance of an image intensifier was clinically preferred to buttress plate fixation for the treatment of non-displaced tibial plateau fractures [22, 23]. In contrast, displaced fractures of the medial plateau (Schatzker type IV) often are quite unstable and are generally best treated with open reduction and internal fixation with a medial buttress plate [24]. The results of the present study also proved that the plate-screw fixation to treat displaced medial tibial fractures provided better stability as well as rigidity and should be clinically preferred for the treatment of medial tibial plateau fractures.

There are a few limitations inherent in this study. First, the materials of the cortical and cancellous bone were both imitated and so may not reflect the actual conditions of bone properties. Second, cyclic loading was not simulated because the simulation of dynamic motion of the joints is time-consuming and requires substantial computer resources [8]. Thus, the displacement calculated may be underestimated. Third, the tibiofibular joints were simplified and simulated by bonding the tibia and fibula together, which may not reflect the condition of the actual joint motion.

## Conclusion

This study demonstrated that the load transmission mechanism between plate-screw fixation system and screw fixation system was different and the stability of the plate-screw fixation system was superior to the screw fixation system. Clinically, rigid fixation with plate and screws following anatomic reduction should be preferred for Schatzker type IV fractures.

## Abbreviations

DICOM: Digital Imaging and Communications in Medicine
EVMS: equivalent von Mises stress
ORIF: open reduction and internal fixation
